# Sedative Effects of Dexmedetomidine and Ketamine or Dexmedetomidine and Midazolam on Tear Production, Ocular Perfusion Pressure and Pupil Diameter in Rabbits

**DOI:** 10.1002/vms3.71017

**Published:** 2026-06-01

**Authors:** Maryam Mohammadzadeh, Reza Azargoun, Rahim Mohammadi

**Affiliations:** ^1^ Faculty of Veterinary Medicine Department of Internal Medicine and Clinical Pathology Urmia University Urmia Iran; ^2^ Faculty of Veterinary Medicine Department of Surgery and Diagnostic Imaging Urmia University Urmia Iran

**Keywords:** blood pressure, glaucoma, intraocular pressure, ophthalmology, sedative

## Abstract

**Background:**

Administration of sedatives may be necessary for anxious and uncooperative animals. However, different sedative combinations may have confounding effects on ocular parameters. These effects can be crucial in ophthalmology.

**Objectives:**

This study aims to investigate the effects of sedation with dexmedetomidine and ketamine, or dexmedetomidine and midazolam, on tear production (TP), ocular perfusion pressure (OPP) and pupil diameter (PD) in fourteen male New Zealand rabbits.

**Methods:**

The animals were randomly divided into two equal groups. In the DK group, a combination of dexmedetomidine (0.025 mg/kg) and ketamine (30 mg/kg), and in the DM group, a combination of dexmedetomidine (0.1 mg/kg) and midazolam (2 mg/kg) was administered. TP, OPP and PD were measured 15 min before administration of the sedatives, as well as 5, 15 and 20 min after.

**Results:**

Both sedative protocols induced a significant decrease in TP and OPP (*p* < 0.05), but no significant change was observed in the PD (*p* > 0.05). While the mean TP did not significantly differ between the two groups, the DK group showed considerably lower mean OPP and higher mean PD compared to the DM group.

**Conclusions:**

According to our results, using artificial tears following both prescribed sedative protocols seems necessary. However, we recommend that dexmedetomidine‐ketamine not be administered in rabbits at risk for glaucoma, as this protocol can significantly reduce OPP. Otherwise, the mentioned protocol can be a good choice in situations where fundoscopy is required, as it does not lead to miosis in healthy rabbits.

## Graphical abstract

Sedative effects of intramuscular injection of dexmedetomidine and ketamine or dexmedetomidine and midazolam on tear production (TP), ocular perfusion pressure (OPP) and pupillary diameter (PD) in two groups of seven male New Zealand rabbits (G1 and G2, respectively). The asterisk indicates a statistically significant difference compared to the other group (*p* < 0.05).

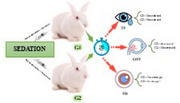

## 1 Introduction

Rabbits are of great interest as pets and laboratory animals, especially in ophthalmological research (Ghaffari and Moghaddassi 2010). However, these animals may become easily stressed and uncooperative, challenging ophthalmological examinations. As rabbits' mortality associated with general anaesthesia is high compared to dogs and cats (1.39% vs. 0.17% and 0.24%, respectively), sedatives can be used to reduce animals' stress and facilitate routine clinical diagnostic techniques (Aghababaei et al. 2021; Bellini et al. 2014; Yavuz et al. 2021). Although sedation can induce adverse ophthalmic side effects, it has been documented that a combination of sedatives may minimize adverse effects (Aghababaei et al. 2021; Di Pietro et al. 2021; Karamichali et al. 2020). As no sedative agent has yet met all the requirements of ophthalmic procedures, research continues to find an ideal sedation protocol with the least adverse effects on ocular parameters (Rastabi et al. 2018).

Dexmedetomidine is a potent alpha‐2 adrenergic agonist with sedative, analgesic and anxiolytic properties, as well as limited adverse side effects (Di Pietro et al. 2021). However, bradycardia and bradypnea were observed in the rabbits that were given dexmedetomidine, presumably caused by decreased sympathetic tone and CNS depression. Therefore, to overcome these reactions, dexmedetomidine is usually prescribed together with other agents (González‐Gil et al. 2015). Ketamine, as a dissociative anaesthetic, antagonizes *N*‐methyl‐d‐aspartate receptors, and it is used in rabbits due to its rapid action with minimal depressant effects on the cardiovascular and respiratory systems. Midazolam, a benzodiazepine receptor agonist, has muscle‐relaxant and calming properties with minimal cardiorespiratory depressant effects in rabbits (Bellini et al. 2014; Yavuz et al. 2021).

In ophthalmic examinations, evaluation of tear production (TP) and intraocular pressure (IOP) is important as they provide invaluable diagnostic and management clues to various ophthalmologic conditions (Cinar et al. 2024). Tears on the ocular surface have antibacterial and antinociceptive properties and provide an ideal extracellular environment for the corneal and conjunctival epithelium. Insufficient TP can cause inflammation of the cornea and conjunctiva (Paolini et al. 2024). An increase in IOP is known as a modifiable risk factor for glaucoma. Nevertheless, glaucomatous injuries still develop in many patients, sometimes despite a notable reduction in IOP. This suggests that other factors may be involved (Ch'ng et al. 2021). It seems that disrupted regulation of ocular blood flow also plays a role in some ophthalmic pathologic processes, such as glaucoma. So, an increase in IOP and a decrease in systemic blood pressure may cause low ocular perfusion pressure (OPP), subsequently leading to ocular hypoxia and/or ischemia (Schmidl et al. 2011). Ideally, agents that increase pupil diameter (PD) without significant efficacy on IOP would be a good choice for posterior eye examinations and intraocular procedures (Schroder et al. 2018).

We hypothesized that different sedative combinations may have confounding effects on ocular parameters. These effects should be considered when choosing a sedation protocol and interpreting clinical findings to avoid inaccurate results. Therefore, this study aimed to evaluate the effects of dexmedetomidine with ketamine and dexmedetomidine with midazolam, as two different sedation protocols, on TP, OPP and PD in healthy rabbits.

## 2 Materials and Methods

This study was conducted after obtaining approval from the Animal Ethics Committee of Urmia University. Fourteen male New Zealand rabbits, 1.53 ± 0.45 years old and weighing 2.4 ± 0.3 kg, participated in this study. The rabbits were considered healthy based on complete physical and ophthalmic examinations and the evaluation of routine blood parameters before beginning this research. The ophthalmic examination included assessing the pupillary light reflex, dazzle reflex, Schirmer tear test (PSM, India), direct ophthalmoscopy (Welch Allyn, USA), tonometry (Riester's Schiötz tonometer, Germany) and fluorescein staining (Toosnegah, Iran). Animals showing any signs of disease or ophthalmic abnormalities, such as blepharospasm, dry eye, ocular discharge, conjunctivitis, red eye, corneal ulcers, ocular opacity, uveitis, abnormal IOP and cataracts, were excluded from the study. To acclimate the rabbits to the experimental environment before the beginning of the study, they were kept in individual cages with controlled environmental conditions for 2 weeks and fed with hay, pellets and ad libitum water.

Initially, the rabbits were transferred to the examination table and given 10 min to calm down while kept in a cage. An experienced clinician in veterinary ophthalmology performed the ophthalmic examinations. Due to the importance of performing the Schirmer tear test before any other ophthalmological procedures and the necessity of topical anaesthesia of the eye to measure IOP, the Schirmer tear test II (STT‐II) was used in this study. The STT‐II was performed according to the study of Selk Ghaffari et al. (2009) to evaluate TP. Therefore, after applying one drop of topical anaesthesia (0.5% Tetracaine, Darou Pakhsh, Iran), one sterile strip was placed in the lower conjunctival fornix of each eye, and the wetness value was recorded after one minute (Selk Ghaffari et al. 2009). The IOP was measured with a pre‐calibrated Schiotz Tonometer. Therefore, the rabbits were kept in a lateral recumbent position using a towel wrapped around them. Then, the eyelids were gently retracted without applying pressure to the globe, and the tonometer was positioned perpendicular to the centre of the cornea. The scale value was converted using the conversion table supplied with the instrument to the IOP value in millimetres of mercury (Ali et al. 2014). The mean of three readings was recorded each time (Abdulsahib and Abood 2021). The respiratory rate (RR) was measured by counting chest movements. Additionally, the heart rate (HR), peripheral haemoglobin oxygen saturation (SpO_2_), mean arterial pressure (MAP) and rectal temperature (RT) of the rabbits were recorded using a multi‐parameter veterinary patient monitoring device (PM‐7000VET, Zoncare, China) (Cinar et al. 2024). So, the OPP was calculated by the measured MAP and IOP using the following formula: OPP = MAP − IOP (Costa et al. 2016). A Castroviejo calliper (Mark, Pakistan) was adjusted based on the pupil's diameter adjacent to the cornea's surface to measure the PD (Aghababaei et al. 2021).

The TP, OPP and PD were measured before (−15 min, T0), and 5 (T1), 15 (T2) and 20 (T3) minutes after administration of the sedatives in both eyes of the rabbits (Ghaffari and Moghaddassi 2010). For sedation, in the DK group (G1), the combination of dexmedetomidine (Dechra, USA) (0.025 mg/kg) and ketamine (Bremer, Germany) (30 mg/kg), and in the DM group (G2), the combination of dexmedetomidine (0.1 mg/kg) and midazolam (Exir, Iran) (2 mg/kg) was administered in the same syringe intramuscularly (Cardoso et al. 2020; Yanmaz et al. 2022). To avoid errors, the parameters were measured between 9:00 and 11:00 AM by an operator blinded to the sedation protocols and in the animals with the same position. During the investigations, the rabbits were gently physically restrained on a heating pad to inhibit hypothermia.

The statistical analysis of the obtained data was done with SPSS version 20.0. The Shapiro–Wilk test was used to assess the normality of the data. A paired‐sample *t*‐test was used to compare the statistical differences in values between the right and left eyes. Repeated measures analysis of variance and the Bonferroni test were used to assess the values within a group. Also, the independent sample *t*‐test was performed to assess the differences between the groups. A *p* value < 0.05 was accepted as statistically significant. The results are shown as mean ± SD.

## 3 Results

All rabbits remained sedated for 20 min and completed the experimental procedures without any sedation‐related complications, and no antagonists were used. The total duration of sedation (minutes) was considerably greater in the DK group (61.42 ± 5.41) than in the DM group (49.57 ± 6.24) (*p* < 0.05). The changes in HR, RR, SpO_2_ and RT are summarized in Table 1. There was no statistically significant difference in TP, IOP, OPP and PD values of the left and right eyes, so the mean values of these parameters in the two eyes were considered for analysis.

**TABLE 1:** Mean ± SD of HR (beats/minute), RR (breaths/minute), SpO_2_ (%) and RT (°C) values in the rabbits, 15 min before (T0), and 5 (T1), 10 (T2) and 20 (T3) minutes after administration of dexmedetomidine and ketamine (G1) or dexmedetomidine and midazolam (G2).

		Pre‐sedation	Post‐sedation		
		T0	T1	T2	T3
G1	HR	231 ± 27^a^	206 ± 29^ab^	197 ± 25^b^	197 ± 24^b^
	RR	77 ± 12^Aa^	50 ± 10^Ab^	54 ± 11^Aab^	55 ± 8^Aab^
	SpO_2_	96 ± 1^a^	87 ± 1^b^	85 ± 2^b^	89 ± 2^Bb^
	RT	38.8 ± 0.3^a^	38.7 ± 0.3^a^	38.5 ± 0.4^a^	38.6 ± 0.4^a^
G2	HR	207 ± 19^a^	196 ± 21^b^	183 ± 25^c^	182 ± 27^c^
	RR	82 ± 10^Aa^	52 ± 11^Ab^	47 ± 12^Ab^	41 ± 10^Bb^
	SpO_2_	97 ± 0.6^a^	89 ± 4^b^	88 ± 4^b^	93 ± 3^Aa^
	RT	38.5 ± 0.4^ab^	38.3 ± 0.3^a^	38.2 ± 0.4^b^	38.3 ± 0.2^ab^

*Note*: Different uppercase letters indicate statistically significant differences related to each parameter between the groups (*p* < 0.05). Different lowercase letters indicate statistically significant differences related to each parameter within a group (*p* < 0.05).

The results of TP, MAP, IOP, OPP and PD changes are presented in Tables 2, 3, 4. Sedation in both groups caused a significant and relatively stable decrease in TP. However, at the third evaluation time, no statistically significant difference was observed in the rabbits of the DM group compared to the time before sedation. TP did not show a statistically significant difference between the two studied groups at different times (*p* > 0.05).

**TABLE 2:** Mean ± SD of TP values (mm/min) in the rabbits, 15 min before (T0), and 5 (T1), 10 (T2) and 20 (T3) minutes after administration of dexmedetomidine and ketamine (G1) or dexmedetomidine and midazolam (G2).

	Pre‐sedation	Post‐sedation		
	T0	T1	T2	T3
G1	8.57 ± 2.63^a^	6.14 ± 2.03^b^	5.00 ± 2.16^b^	5.85 ± 2.11^b^
G2	7.85 ± 2.19^a^	4.71 ± 1.88^b^	3.57 ± 1.71^b^	4.85 ± 1.06^ab^

*Note*: Different letters indicate statistically significant differences within a group (*p* < 0.05).

**TABLE 3:** Mean ± SD of MAP, IOP and OPP values (mm Hg) in the rabbits, 15 min before (T0), and 5 (T1), 10 (T2) and 20 (T3) minutes after administration of dexmedetomidine and ketamine (G1) or dexmedetomidine and midazolam (G2).

		Pre‐sedation	Post‐sedation		
		T0	T1	T2	T3
G1	MAP	83.14 ± 5.63^aA^	73.00 ± 5.71^bA^	60.14 ± 4.87^cA^	58.28 ± 4.88^cB^
	IOP	15.57 ± 2.69^aA^	17.28 ± 2.42^bA^	19.28 ± 2.49^bA^	18.42 ± 1.51^abA^
	OPP	67.57 ± 3.64^Aa^	55.71 ± 4.82^Ab^	40.85 ± 4.22^Bc^	39.85 ± 4.48^Bc^
G2	MAP	81.42 ± 7.76^aA^	70.71 ± 6.23^bA^	63.42 ± 6.26^cA^	67.14 ± 5.87^dA^
	IOP	14.71 ± 3.81^aA^	12.85 ± 3.02^bB^	13.14 ± 2.19^abB^	14.57 ± 2.93^aB^
	OPP	66.71 ± 5.79^Aa^	57.85 ± 5.08^Ab^	50.28 ± 6.12^Ac^	52.57 ± 4.19^Ac^

*Note*: Different uppercase letters indicate statistically significant differences related to each parameter between the groups (*p* < 0.05). Different lowercase letters indicate statistically significant differences related to each parameter within a group (*p* < 0.05).

**TABLE 4:** Mean ± SD of PD values (mm) in the rabbits, 15 min before (T0), and 5 (T1), 10 (T2) and 20 (T3) minutes after administration of dexmedetomidine and ketamine (G1) or dexmedetomidine and midazolam (G2).

	Pre‐sedation	Post‐sedation		
	T0	T1	T2	T3
G1	8.28 ± 1.11^A^	8.85 ± 1.21^A^	9.57 ± 0.53^A^	8.85 ± 0.89^A^
G2	7.57 ± 1.81^A^	6.28 ± 1.25^B^	5.57 ± 0.53^B^	6.00 ± 1.00^B^

*Note*: Different letters indicate statistically significant differences between the groups (*p* < 0.05).

In both groups, OPP decreased significantly after sedation, so that in the 20th minute after sedation, it was still lower than the values before administration of drugs. However, in the last evaluation stage, OPP in the DM group was significantly higher than in the DK group (*p* < 0.001).

Although PD showed increasing (DK group) and decreasing (DM group) changes compared to the time before sedation, they were not statistically significant. In post‐sedation evaluations, the reduction of PD in rabbits of the DM group was significant compared to the DK group (*p* < 0.01).

## 4 Discussion

Sedatives are routinely used to calm animals for ophthalmic examinations; nonetheless, these may lead to adverse ophthalmic side effects (Erol et al. 2021; Raušer et al. 2022). This study showed that both sedative combinations can reduce TP and OPP in rabbits. However, the decrease in tears in the group sedated with dexmedetomidine and midazolam was less stable (less than 20 min). Although the induction of sedation did not affect the PD of the rabbits, post‐sedation evaluations showed a significant decrease in the PD of the group receiving the combination of dexmedetomidine and midazolam compared to the other group.

It should be noted that the doses of dexmedetomidine used in this study were not identical. Therefore, differences in sedative effects between the study groups are conceivable. However, despite the lower dose of dexmedetomidine in the DK group, the duration of sedation was significantly longer, which could be related to the effects and dosage of ketamine.

Tear secretion is important to maintain eye health as well as prevent corneal dryness and exposure keratopathy (Bulut and Yaygingul 2024; Di Pietro et al. 2021). A transient decrease in TP can be expected following the administration of various sedative drugs, which may be attributed to the central effects of these agents on the neural regulation of TP and/or disruption of blinking leading to increased tear evaporation (Aghababaei et al. 2021; Paolini et al. 2024). It has been documented that different dexmedetomidine sedation protocols can considerably reduce TP in dogs, cats and black‐tailed prairie dogs (Aghababaei et al. 2021; Di Pietro et al. 2021; Paolini et al. 2024; Roberts et al. 2019). Similar to these studies, although our results also showed that both sedation protocols caused a significant TP reduction, these changes were approximately within the reference range reported in rabbits (5 ± 3 mm/min) (Selk Ghaffari et al. 2009). According to the study of Di Pietro et al. (2021), tear hypoproduction caused by dexmedetomidine can be related to mechanisms such as central effects, vasoconstriction of the lacrimal gland, metabolic changes and nociception. The first mechanism can be the direct effect of central regulation carried out through the autonomic nervous system. The lacrimal gland is innervated by the lacrimal nerve, a branch of the trigeminal nerve, which has both parasympathetic and sympathetic components. Alpha‐2 agonist sedatives, such as dexmedetomidine, are indicated to inhibit the sympathetic system. Postsynaptic activation of alpha‐2 adrenergic receptors in the central nervous system can reduce basal TP. The next mechanism is systemic vasoconstriction, decreased HR and low cardiac output caused by alpha‐2 agonists. As a result, dexmedetomidine can reduce lacrimal gland perfusion and tear secretion. Metabolism changes at the cellular level of the lacrimal gland through the alpha‐2 adrenergic receptor have also been suggested to reduce tear secretion. In addition, it is also thought that increased alpha‐2 adrenergic‐mediated anti‐nociception reduces reflex tear secretion (Di Pietro et al. 2021).

A more significant decrease in TP may occur when alpha‐2 agonists are administered with other sedatives or anaesthetics (Raušer et al. 2022). This may explain the non‐statistical decrease of TP in the DM group compared to the DK group. Considering the slight cardiovascular effect of midazolam on cats, it can be assumed that this drug has a mild impact on TP in rabbits as well. Furthermore, the combination of synergistic drugs can also intensify tear hypoproduction (Paolini et al. 2024). In dogs, it has been documented that a combination of midazolam and an alpha‐2 agonist has a synergistic effect, whereas midazolam as a sole agent shows weak sedative properties (Khaleghi et al. 2024). Therefore, it can be considered that the combination of midazolam with dexmedetomidine has a synergistic effect on rabbits' TP. On the other hand, ketamine has been found to increase TP in dogs, which could be due to stimulation of parasympathetic innervation and increased lacrimal gland blood flow (Raušer et al. 2022). Nevertheless, the administration of ketamine and dexmedetomidine in rabbits of the DK group reduced TP, which may be due to the masking of the effects of ketamine by dexmedetomidine. Consistent with our results, it was also documented in cats that combining ketamine and medetomidine significantly reduced TP (Paolini et al. 2024). The non‐statistical increase in TP in the last stage of the study in both groups could possibly be due to the existence of a compensatory mechanism. For example, previous studies have demonstrated that the conjunctival epithelium of rabbits can provide sufficient tear volume (Bhattacharya et al. 2015).

The difference between an organ's arterial and venous blood pressure can determine its perfusion pressure. Assuming that MAP represents the mean ocular arterial pressure and IOP represents the mean ocular venous pressure, the difference between MAP and IOP estimates the OPP. Any decrease in MAP or increase in IOP can reduce the OPP (Abdulsahib and Abood 2021; Ch'ng et al. 2021). However, because blood pressure is more intense than ocular pressure, OPP is likely to be more sensitive to MAP fluctuations than IOP. It has been documented that reduced OPP is a substantial risk factor for higher incidence and development of glaucoma. In addition, low OPP can increase the vulnerability of the optic disc through its hypoperfusion, which causes deprivation of oxygen and nutrition to the optic nerve head (Kim et al. 2020).

It is worth noting that ocular blood flow is specified not only by OPP but also via vascular resistance. Blood flow regulation may occur through vasodilation or vasoconstriction, independent of OPP variations. In retinal circulation, auto‐regulation of blood flow is desirable because the metabolic function of the retina and its subsequent need for nutrients, oxygen and elimination of waste products depend on blood flow. Interestingly, unlike humans, the rabbit retina has negligible circulation and is nourished mainly by the choroid. However, the choroid is not over‐perfused to oxygenate the retina, and the inner parts of the photoreceptors may become hypoxic following a slight decrease in choroidal blood flow. Also, choroidal blood flow will stop when OPP is zero (Caprioli and Coleman 2010). The mean OPP value in healthy New Zealand White rabbits was reported by Costa et al. to be 68.85 mm Hg (Abdulsahib and Abood 2021).

Alpha‐2 agonists have dose‐dependent clinical effects (Paolini et al. 2024). Dexmedetomidine can alter MAP in a dose‐dependent fashion, with a lower dose of dexmedetomidine causing a greater decrease in MAP (Yavuz et al. 2021). Moreover, it has been reported that alpha‐2 agonists reduce IOP by constricting ciliary body vessels, decreasing ciliary body blood flow and reducing aqueous humour production (Khaleghi et al. 2024). In the present study, sedation in both groups resulted in a considerable reduction in MAP compared to the time before medication administration. Accordingly, a decrease in OPP was expected in all rabbits. However, based on what was mentioned about the dose‐dependent effects of alpha‐2 agonists, the reduction in MAP and subsequently OPP was more pronounced in the DK group than in the DM group, which is probably due to the lower dose of dexmedetomidine administered in the DK group. On the other hand, IOP changes in the studied groups showed a completely different trend, with a significant increase and decrease observed after sedation induction in the DK and DM groups, respectively. The difference in IOP could possibly explain the greater reduction in OPP in the DK group compared to the DM group. It is stated that midazolam, as a sole agent, shows no effects on IOP, while ketamine can increase IOP. These findings can be attributed to the relaxation of extraocular muscles caused by midazolam and the increase in extraocular muscle tone by ketamine (Pierce‐Tomlin et al. 2020). It is worth noting that in one study, neither diazepam nor acepromazine could reliably prevent ketamine‐induced IOP elevation in healthy rabbits (Ghaffari and Moghaddassi 2010). In our study, although dexmedetomidine failed to prevent ketamine‐induced IOP elevation, the increased IOP was clinically insignificant.

Previously, the mean ± SD of PD in healthy, unsedated mixed‐breed rabbits was reported to be 7.7 ± 2.1 (Kovalcuka and Nikolajenko 2020). In our study, none of the protocols significantly changed the rabbits' PD compared to the time before sedation. However, the mean of PD in the DM group was significantly lower than in the DK group. It has been documented in dogs that the injection of dexmedetomidine can change PD in a decreasing trend (Aghababaei et al. 2021). Although, according to the authors' knowledge, no study has been conducted on the combined effects of dexmedetomidine and midazolam on rabbits' PD, perhaps the proven synergistic effects of these drugs also added to the miotic properties of dexmedetomidine and reduced the PD in the DM group. The increase in PD in rabbits in the DK group can probably be attributed to ketamine. Because it has been documented that ketamine increases PD in dogs (Kovalcuka et al. 2013). However, we could not find any information about the mechanism of this possible effect of ketamine in the literature review.

In the present study, several limitations and differences compared to other studies should be clarified. One was the different sedation protocols (in terms of administered medications and dosages) and the lack of assessing sedation scales, which made effective comparisons challenging, although our goal was not an anaesthesiology study. Another was the short duration of the measurements, which may not have fully revealed the potential pharmacological effects of the drugs. However, short‐term sedation is usually used for most ophthalmic examinations, and the clinician should be able to interpret the clinical findings correctly at this time, taking into account the side effects of sedatives. IOP is another issue, which was measured in this study using a Schiotz Tonometer. Since there is no gold standard in veterinary medicine, all available tonometers can be used. Today, despite the invention of advanced instruments, the Schiotz Tonometer is still used because of its affordability and ability to provide reliable, reproducible results with good correlation (Aghababaei et al. 2021; Wrześniewska et al. 2018). Finally, OPP actually must be characterized by the difference between the arterial and venous pressure at the inlet and outlet of the eye, respectively. However, currently available facilities cannot directly measure this parameter without invasiveness and anaesthesia. Previous studies documented that TP and IOP in animals may be affected by various factors such as age, breed, gender, body weight, daytime, season and methodology (Cinar et al. 2024; Erol et al. 2021). Furthermore, our study was performed on healthy rabbits, so it can be assumed that even using the same protocol, different results may be obtained in rabbits with various ophthalmic diseases.

## 5 Conclusion

This study confirmed that both dexmedetomidine‐ketamine and dexmedetomidine‐midazolam sedation protocols can significantly reduce tear secretion in rabbits. Therefore, using artificial tear compounds even for short‐term sedation seems necessary to prevent corneal damage. We also strongly recommend not using dexmedetomidine‐ketamine sedation in rabbits at risk of glaucoma. According to the significant decrease in OPP observed in the DK group, this sedation protocol may accelerate glaucoma progression and glaucomatous damage in susceptible rabbits. Otherwise, due to the lack of miosis induction in the rabbits of the DK group, sedation with dexmedetomidine‐ketamine can be a suitable choice in situations where a funduscopic examination is needed or anterior synechiae should be prevented in rabbits with uveitis.

## Author Contributions

**Maryam Mohammadzadeh**: project administration, resources, writing – review and editing. **Reza Azargoun**: investigation, methodology, writing – review and editing. **Rahim Mohammadi**: visualization, writing – original draft, formal analysis.

## Funding

Financial support was provided by the vice chancellor of Research Council of Urmia University.

## Ethics Statement

The Animal Ethics Committee of Urmia University approved the experiments, and their guidelines were followed (Code number: IR‐UU‐AEC‐3/60).

## Conflicts of Interest

The authors declare no conflicts of interest.

## Data Availability

All data in this study are included in this published article.
